# G6PC1 expression as a prognostic biomarker associated with metabolic reprogramming and tumor microenvironment in hepatocellular carcinoma

**DOI:** 10.3389/fimmu.2025.1623315

**Published:** 2025-08-01

**Authors:** Xilong Tang, Jianjin Xue, Xiao Li, Jie Zhang, Jiajia Zhou

**Affiliations:** ^1^ Guangdong Provincial Key Laboratory of Malignant Tumor Epigenetics and Gene Regulation, Sun Yat-Sen Memorial Hospital, Sun Yat-Sen University, Guangzhou, China; ^2^ Department of Surgery, Sun Yat-Sen Memorial Hospital, Sun Yat-Sen University, Guangzhou, China

**Keywords:** G6PC1, hepatocellular carcinoma, prognostic biomarker, metabolic reprogramming, tumor microenvironment

## Abstract

**Background:**

Hepatocellular carcinoma (HCC) is the most prevalent primary liver cancer, characterized by a poor prognosis. Many HCC patients are diagnosed at an advanced stage due to the lack of reliable prognostic biomarkers. G6PC1 (Glucose‐6‐Phosphatase Catalytic Subunit 1) is abnormally expressed in various cancers, including HCC. This study aimed to investigate the biomarker potential and biological functions of G6PC1 to elucidate its impact on HCC pathogenesis.

**Methods:**

G6PC1 expression levels were assessed using TCGA and GEO datasets. Prognostic implications were explored through Kaplan-Meier survival analysis. Potential regulatory transcription factors (TFs) were identified using four prediction tools, and functional mechanisms were investigated via GO and KEGG enrichment analyses. Associations between G6PC1 and HCC metabolic reprogramming, as well as the tumor microenvironment were analyzed.

**Results:**

G6PC1 exhibited low expression levels in HCC, which correlated with poor patient prognosis. HNF4A may act as a regulatory factor for G6PC1 in HCC. Functional analysis identified co-expressed genes associated with metabolism-related pathways. Furthermore, G6PC1 was implicated in metabolic reprogramming, immune infiltration, and immunotherapy response.

**Conclusion:**

Low G6PC1 expression, associated with poor HCC prognosis, is a potential prognostic biomarker. Integrated multi-omics analyses underscore its clinical significance, involvement in metabolic reprogramming, and immunomodulatory functions, providing a foundation for further investigation into its prognostic potential and mechanistic contributions in HCC.

## Graphical Abstract

Flowchart of the study design.

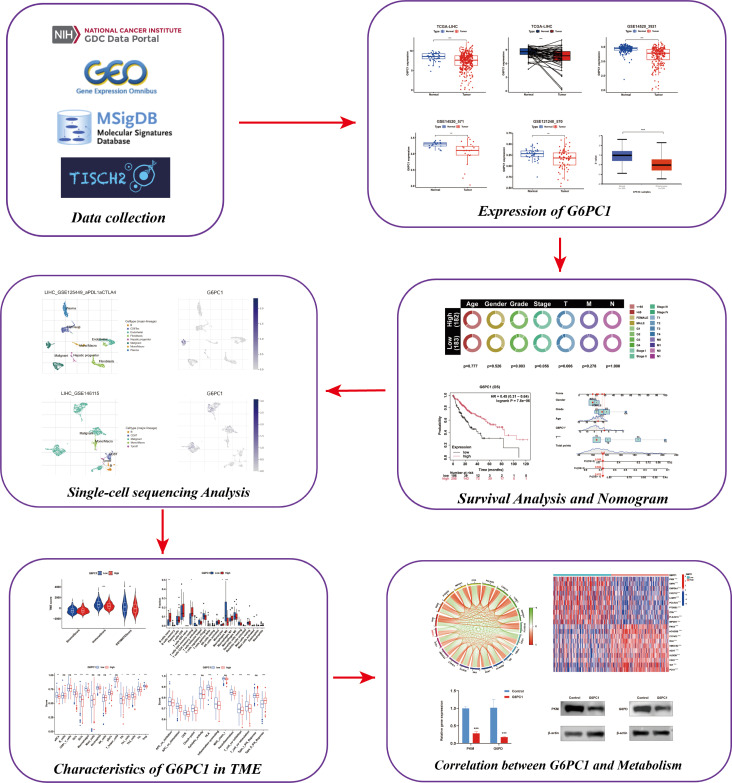

## 1 Introduction

Hepatocellular carcinoma, representing nearly 80% of primary liver malignancies (1), stands as one of the most prevalent digestive system cancers worldwide. With a global incidence ranking fifth and mortality third among cancers (2), HCC presents significant clinical challenges. Its insidious onset and aggressive metastatic potential often delay early diagnosis and intervention (3). While current therapeutic approaches encompass surgical resection, transplantation, ablation, arterial chemoembolization, and emerging targeted and immunotherapies (4), patient outcomes remain suboptimal, as evidenced by disappointing five-year survival rates. Additionally, most HCC patients gained their diagnosis at an advanced stage, missing the opportunity for radical therapy due to diagnostic limitations (5). This underscores the critical demand for novel biomarkers to improve early detection accuracy and prognostic assessment in HCC management.

Glucose-6-phosphate (G6P) is the first intermediate of glucose metabolism linking crucial metabolic pathways such as glycolysis, glycogenesis, *de novo* lipogenesis, and the pentose phosphate pathway (6). G6PC1 is a protein-coding gene that is predominantly expressed in the liver and kidney, with minimal expression in the intestine and pancreas (7). This enzyme catalyzes the conversion of G6P to glucose and inorganic phosphate, representing the final step in the catalytic pathways of gluconeogenesis and glycogenolysis, thereby playing a crucial role in maintaining fasting blood glucose levels (8). In humans, the inheritance of deleterious G6PC1 mutations in both alleles causes the autosomal recessive disease GSD-Ia. In GSD-Ia, deficient G6PC1 activity causes the accumulation of glycogen and fat in the liver and kidneys, and prevents hepatic glucose production by gluconeogenic organs, leading to hypoglycemia (9).

G6PC1 is abnormally expressed in various cancer types, contributing to metabolic reprogramming, proliferation, invasion, and metastasis of tumor cells. Some studies have reported that G6PC1 is highly expressed in ovarian cancer, where it is significantly associated with short-term recurrence and poor prognosis (10). In addition, other studies have reported miRNA-mediated deregulation of G6PC1 expression in HCC (11) and its reduced expression in different gluconeogenic tumor tissues, such as clear cell renal cell carcinoma (12). Research has shown that G6PC1 catalyzes the last step of glycogenolysis, is frequently downregulated to augment glucose storage in pre-malignant cells, driving liver tumor initiation (13). Kim et al. reported that GSD-Ia mice with less than 2% of normal hepatic G6pc activity have an increased risk of HCC development, highlighting the crucial role of this gene in hepatocarcinogenesis (14).

Although previous studies have reported G6PC1 deregulation in HCC and its role in liver metabolism, the impact of G6PC1 on HCC prognosis, as well as its functional significance in the tumor immune microenvironment and metabolic reprogramming, remains unclear. Our research focuses on the effects of altered G6PC1 expression on HCC oncogenic mechanisms, metabolic reprogramming, and immune regulatory role. Initially, we performed a comprehensive analysis of public datasets. The results revealed that consistent downregulation of G6PC1 across multiple tumor types, with its reduced expression correlating significantly with adverse clinical outcomes. Systematic evaluation of clinicopathological parameters demonstrated the prognostic relevance of G6PC1 expression patterns. Furthermore, we characterized the genomic alterations and epigenetic modifications affecting G6PC1 while investigating its influence on immune microenvironment composition. Single-cell RNA sequencing analysis identified predominant G6PC1 expression in both hepatocytes and malignant cells. Functional and pathway enrichment analysis highlighted G6PC1’s involvement in HCC metabolic reprogramming. The results offer crucial insights into identifying G6PC1 as a tumor suppressor, highlighting its close association with immunity and metabolism. Overall, we utilized multi-omics datasets from the GEO, TCGA, HCCDB, TISCH, and HPA databases to comprehensively explore the impact of G6PC1 on HCC prognosis, metabolic reprogramming, tumor immune microenvironment, drug sensitivity, and immunotherapy potential.

## 2 Materials and methods

### 2.1 Data collection

The TIMER2.0 database enables the analysis of differential gene expression between tumor and control tissues using data from the TCGA database (15). This tool was utilized to assess G6PC1 expression across various cancers. Transcriptomic profiles and corresponding clinical data from HCC specimens and matched adjacent non-tumorous liver tissues were obtained from the TCGA, with RNA-seq data normalized to TPM format. Additionally, mRNA expression data from the GSE14520_3921, GSE14520_71, and GSE121248_570 datasets of the GEO database were employed for external validation of G6PC1 expression differences. Variations in G6PC1 protein expression between HCC cells and normal hepatocytes were evaluated using data from the UALCAN website (16). Immunohistochemical data for G6PC1 in HCC specimens were obtained from the HPA database (17).

### 2.2 Survival analysis and construction of a nomogram

The associations between clinical features and survival were evaluated using univariate and multivariate Cox regression analyses, with significant parameters from the univariate analysis included in the multivariate model (18). The Kaplan-Meier plotter database, an online platform for conducting survival analyses (19), was utilized to assess the prognostic significance of G6PC1 in HCC patients across varying levels of immune cell infiltration. Additionally, a nomogram was developed, integrating G6PC1 expression levels and clinical parameters to predict the probabilities of 1-, 3-, and 5-year survival. The predictive accuracy of this nomogram was assessed using calibration curves (20).

### 2.3 Single-cell and spatial transcriptome analysis of G6PC1

To investigate the expression of G6PC1 in the tumor microenvironment (TME) of HCC at a single-cell level, we utilized the Tumor Immune Single-cell Hub 2 (TISCH2) database, the Monaco database, and the HCCDB database (21, 22). Additionally, we explored the spatial expression of G6PC1 in HCC using data from the HCCDB database.

### 2.4 Identification of differentially and co-expressed genes

We utilized Pearson’s correlation analysis to identify genes that are co-expressed with G6PC1 in TCGA-LIHC (23). Patients in the TCGA dataset were segregated into G6PC1^low^ and G6PC1^high^ groups based on median G6PC1 expression. Differentially expressed genes (DEGs) in the TCGA-LIHC cohort were detected and visualized using the limma package, with an FDR < 0.05 and |log2FoldChange| > 1 set as the criteria for identifying DEGs. Venn diagrams were constructed to pinpoint the intersections between the DEGs and the co-expressed genes. Functional enrichment analyses were performed on the overlapping genes using the ‘clusterProfiler’ package (24). For the GSEA analysis, the gene sets “c2.cp.kegg.v7.4.symbols.gmt” and “c5.go.v7.4.symbols.gmt” were employed (25).

### 2.5 Gene mutations and DNA methylation analysis

G6PC1 mutations and copy number variations (CNVs) were analyzed using cBioPortal to assess their association with HCC prognosis (26). Mutation subtypes were further characterized using the COSMIC database. DNA methylation patterns were evaluated via MethSurv (https://biit.cs.ut.ee/methsurv/) and the Shiny Methylation Analysis Resource Tool (SMART; http://www.bioinfo-zs.com/smartapp/) (27, 28).

### 2.6 Immune cell infiltration analysis

The ESTIMATE algorithm, which infers the proportions of immune and stromal cells in tumor samples through gene expression signatures, was used to calculate the ImmuneScore, StromalScore, and ESTIMATEScore for predicting tumor purity (29). Two immune-related algorithms, CIBERSORT and ssGSEA, were employed to assess the immune landscape and activity across G6PC1^low^ and G6PC1^high^ groups (30, 31). Comparative analysis of immune cell infiltration patterns between G6PC1^high^ and G6PC1^low^ groups was performed using the nonparametric Wilcoxon rank-sum test. To further explore the relationship between G6PC1 and immune cells, seven algorithms—XCELL, TIMER, QUANTISEQ, MCPCOUNTER, EPIC, CIBERSORT abs, and CIBERSORT (32).

### 2.7 Role of G6PC1 in immunotherapy

The study evaluated the correlation between G6PC1 expression levels and 48 genes associated with immunological checkpoints. Additionally, TIDE scores were obtained from the TIDE website (http://tide.dfci.harvard.edu) to compare differences in TIDE between the G6PC1^low^ and G6PC1^high^ groups (33). Tumor mutation burden (TMB) and microsatellite instability (MSI) scores were also assessed for both patient groups (34). Furthermore, the IMvigor210 cohort, comprising 208 bladder cancer patients treated with anti-PD-L1 therapy, was selected to validate the associations between G6PC1 expression levels and the benefits of immunotherapy (35).

### 2.8 Identification of potential TFs

To explore the regulatory mechanisms underlying G6PC1 expression, four databases—HTFtarget, ChIP_Atlas, GTRD, and ENCODE—were employed to predict potential TFs (36). The predictions from these databases were cross-analyzed to pinpoint key TFs.

### 2.9 Sensitivity analysis of G6PC1 with anticancer drugs

The Genomic Scatter Cancer Analysis (GSCA) platform, which integrates data from 33 cancer types (37), was utilized for genomic cancer research. Drug sensitivity analysis was performed using the “Drug” module of GSCA. The Wilcoxon rank-sum test was applied to assess half-maximal inhibitory concentration values between G6PC1^high^ and G6PC1^low^ groups. Furthermore, gene expression profiles of relevant drug targets were analyzed using the DrugBank database (38).

### 2.10 Molecular docking

The DSigDB online database was used to explore the interactions between G6PC1 and drugs (39). The molecular structures of ligands were obtained from the PubChem database, and the structures of target proteins were retrieved from the PDB database. Molecular docking simulations were then performed using the CB-Dock2 online tool (40).

### 2.11 Relationships between the expression of G6PC1 and metabolic-related genes

A total of 945 metabolically associated genes were identified from the KEGG database (41). The potential relationships between G6PC1 expression and these metabolic-related genes were assessed in the TCGA-LIHC cohort. Additionally, a heatmap was employed to visualize the proportions of metabolic-related genes in samples with high versus low G6PC1 expression.

### 2.12 Cell culture

Human hepatocellular carcinoma cell lines PLC/PRF/5 and Huh-7 were obtained from the Cell Bank of the Chinese Academy of Sciences (Shanghai, China). All cell lines were maintained in a humidified incubator at 37°C with 5% CO2 atmosphere, cultured in DMEM medium (Magne) supplemented with 10% fetal bovine serum (FBS).

### 2.13 Lentivirus infection

The human G6PC1 gene was inserted into the vector to construct G6PC1 lentivirus (LV‐G6PC1) (GenePharma, Shanghai, China), with empty vector (LV-NC) serving as negative control (GenePharma, Shanghai, China). Following the manufacturer’s protocol, PLC/PRF/5 and Huh-7 cells were transduced with either LV-NC or LV-G6PC1. Stable polyclonal populations were selected using 2 μg/mL puromycin, and successful transduction was confirmed by the presence of green fluorescent protein (GFP) signal. Successful establishment of the G6PC1 overexpression model was confirmed through both qRT-PCR and Western blot analyses (42, 43).

### 2.14 CCK-8 assay

Well-grown PLC/PRF/5 and Huh-7 cells in the logarithmic growth phase were plated at a density of 2,000 cells per well in a 96-well plate. At indicated time points (0, 24, 48, 72 h), 10 μL CCK-8 reagent was added to each well and incubated for 1 hour. Absorbance at 450 nm was measured using a microplate reader.

### 2.15 RNA extraction and RT‐qPCR

Total RNA was extracted using TRIzol reagent (Invitrogen) and quantified spectrophotometrically (NanoPhotometer N50). cDNA synthesis was performed using the SureScript First-Strand cDNA Synthesis Kit (Servicebio). Relative mRNA expression of G6PC1, G6PD, and PKM was determined by RT-qPCR using the ΔΔCT method with β-actin as the reference gene. Primer sequences are detailed in **Supplementary Table S1**.

### 2.16 Western blotting analysis

Tumor cell protein samples were lysed in RIPA buffer containing freshly added protease and phosphatase inhibitors. The protein concentration was determined via the BCA assay to obtain a sample with a final concentration of 30 μg/μL for subsequent SDS-PAGE. Equivalent protein quantities were resolved by electrophoresis on a polyacrylamide gel and subsequently electroblotted onto a PVDF membrane. To prevent nonspecific binding, the membranes were treated with 5% skim milk for 2 hours at ambient temperature. Following blocking, the membranes were probed with primary antibodies overnight (24 h) and then incubated with secondary antibodies for 1 hour at room temperature. Protein detection was performed using an enhanced chemiluminescence detection kit (Biosharp, Hefei, China).

### 2.17 Statistical analysis

Statistical analyses were performed using R (version 4.4.1) and GraphPad Prism (version 8.0). For unpaired tissue samples, differences in G6PC1 expression were evaluated using the Wilcoxon rank-sum test, while paired samples were analyzed with the paired t-test. Associations between G6PC1 expression and clinical characteristics were examined using χ² tests, logistic regression, Fisher’s exact test, and Wilcoxon rank-sum tests as appropriate. Correlation analyses were conducted using Spearman’s rank correlation coefficient. Statistical significance was defined as a P value less than 0.05.

## 3 Results

### 3.1 Low expression of G6PC1 in HCC

**Figure 1:**
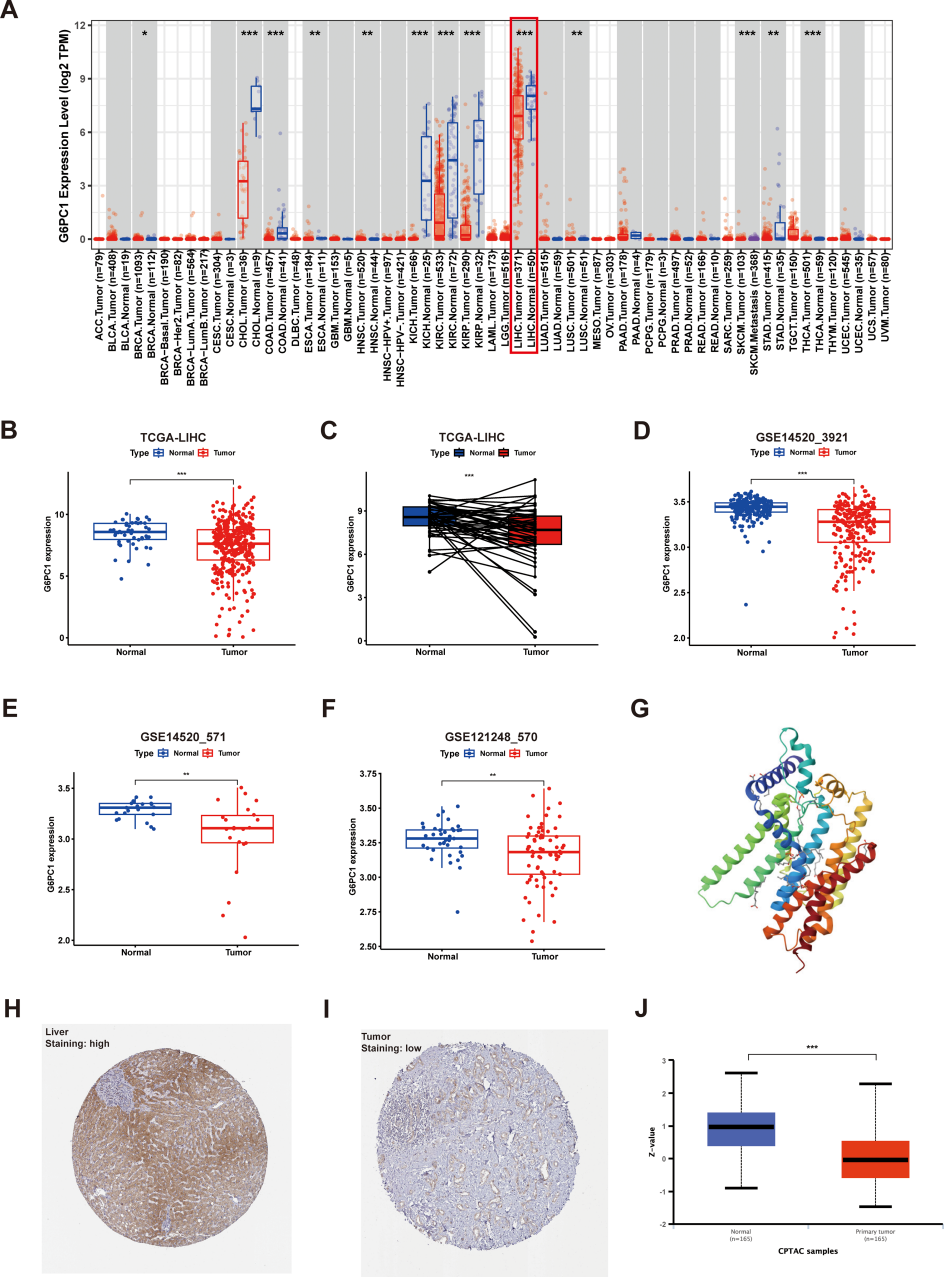
Expression of G6PC1 at the mRNA and protein level. **(A)** G6PC1 expression within various tumors, determined through TIMER2.0. **(B)** TCGA database of HCC and unpaired normal liver tissues. **(C)** TCGA database of HCC and paired normal liver tissues. **(D)** GSE14520_3921. **(E)** GSE14520_571. **(F)** GSE121248_570. **(G)** G6PC1 protein predicted structure. Typical immunohistochemical images of G6PC1 expression in normal liver tissues **(H)** and HCC tissues **(I)** from the HPA database. **(J)** G6PC1 protein expression in HCC tissues and normal liver tissues from the CPTAC database. P values were shown as: *P < 0.05; **P < 0.01; ***P< 0.001.

Initially, we assessed the expression levels of G6PC1 in tumor and control samples using TIMER2.0. Pan-cancer analysis indicated that G6PC1 was significantly downregulated in most cancer types, such as liver cancer, bile duct cancer, colon cancer, kidney clear cell carcinoma, kidney papillary cell carcinoma, and kidney chromophobe (**Figure 1A**). Specifically, G6PC1 expression was considerably lower in patients with HCC than in normal hepatocyte tissues (p<0.001) (**Figure 1B**). In a comparison of 50 pairs of HCC and adjacent non-tumor tissues, a significant reduction in G6PC1 expression was observed in the HCC tissues (**Figure 1C**). This decreased expression of G6PC1 in HCC tissues was also confirmed at the transcriptome level in the GSE14520_3921, GSE14520_571, and GSE121248_570 datasets (**Figures 1D-F**). Furthermore, we retrieved the predicted protein structure of G6PC1 from the HPA database, as illustrated in **Figure 1G**. To further investigate G6PC1 protein expression patterns, immunohistochemical analysis was performed on HCC and normal hepatocyte tissues obtained from the HPA. The results showed that the IHC staining intensity of G6PC1 was significantly lower in HCC tissues than in adjacent non-tumor tissues (**Figures 1H, I**). Additionally, proteomic analysis of the CPTAC dataset further demonstrated significantly reduced G6PC1 protein abundance in HCC specimens relative to normal hepatic controls (**Figure 1J**).

### 3.2 Low G6PC1 expression correlates with adverse clinicopathological features and poor prognosis in HCC

**Figure 2:**
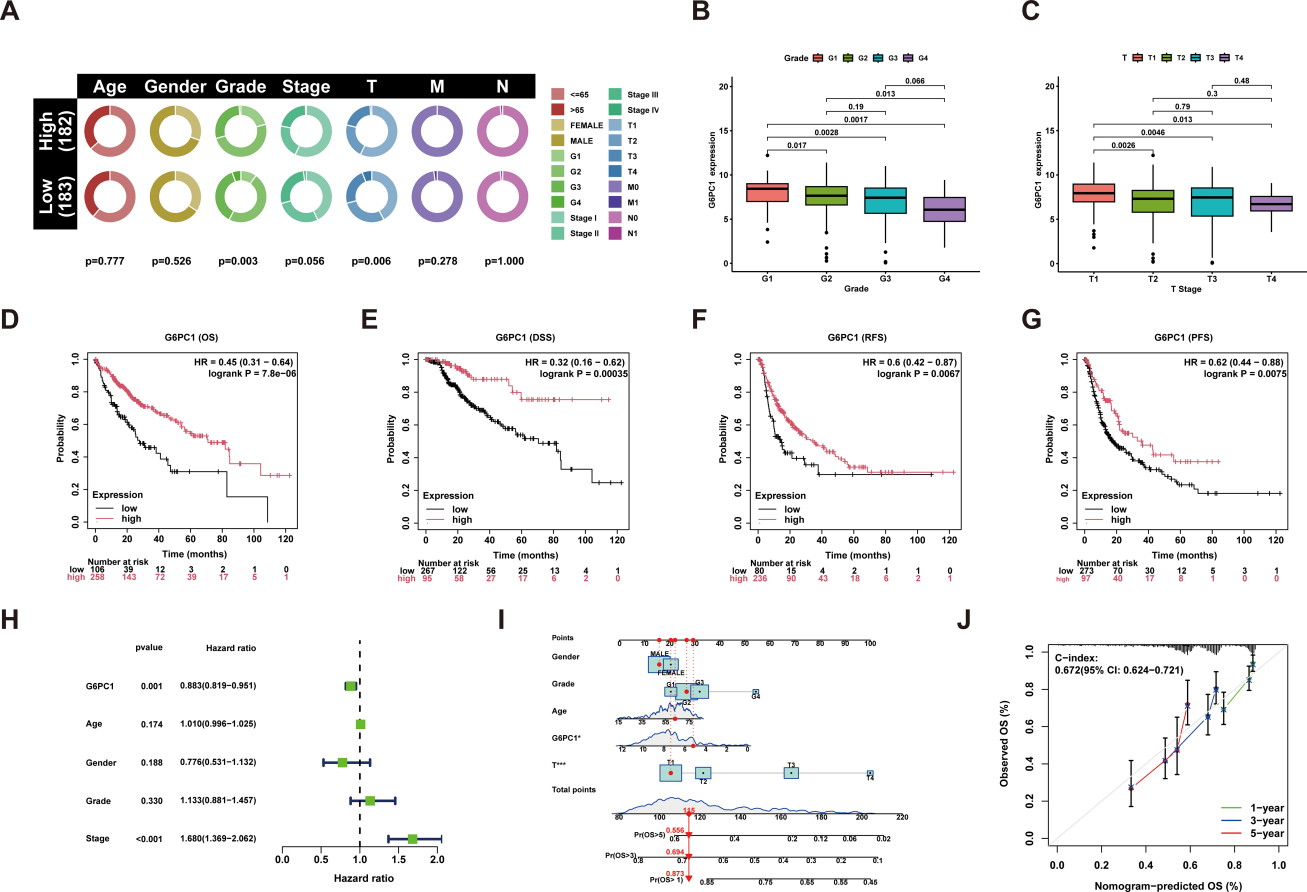
Performance of the G6PC1 for predicting HCC diagnosis and outcomes. **(A)** G6PC1 mRNA levels in relation to clinical features. **(B)** Histologic grade. **(C)** T stage. Kaplan-Meier survival analysis showing OS **(D)**, DSS **(E)**, RFS **(F)** and PFS **(G)** compared to G6PC1 expression. **(H)** Forest map based on univariate Cox analysis for overall survival. **(I)** Prediction of 1-, 3-, and 5-year OS by nomogram. **(J)** Calibration plots were used to validate the nomogram model.

Clinical data and G6PC1 expression levels in HCC patients were sourced from the TCGA database. Univariate analysis was employed to explore the associations between these parameters, revealing a significant correlation between G6PC1 expression and T stage as well as histological grading (**Figure 2A**). Patients with higher tumor grade and advanced T stage exhibited lower G6PC1 expression (**Figures 2B, C**). Survival analysis showed that patients with elevated G6PC1 expression had significantly better overall survival (OS) (HR=0.45, P<0.001) (**Figure 2D**), disease-specific survival (DSS) (HR=0.32, P<0.001) (**Figure 2E**), recurrence-free survival (RFS) (HR=0.6, P<0.01) (**Figure 2F**), and progression-free survival (PFS) (HR=0.62, P<0.01) compared to those with reduced G6PC1 expression (**Figure 2G**). Additionally, univariate Cox regression analysis was conducted to determine whether G6PC1 and clinical-pathological variables could serve as independent prognostic factors for OS. The analysis demonstrated a significant association between G6PC1 and OS (HR = 0.883, 95% CI = 0.819-0.951, P=0.001), and tumor stage was also significantly linked to OS (HR = 1.680, 95% CI = 1.369-2.062, P< 0.001) (**Figure 2H**). To further evaluate G6PC1’s prognostic impact on 1-, 3-, and 5-year survival rates, a nomogram was constructed based on G6PC1 level, age, gender, and T stage. The prognostic nomogram integrated multiple risk factors by calculating a cumulative score for each HCC patient, with higher total scores correlating with worse overall survival outcomes (**Figure 2I**). To evaluate predictive accuracy, calibration analyses demonstrated strong concordance between nomogram-predicted and observed survival probabilities. Notably, the model exhibited superior predictive performance for 1-year survival compared to 3- and 5-year estimates (**Figure 2J**).

### 3.3 The expression of G6PC1 in HCC TME at single-cell and spatial transcriptome levels

**Figure 3:**
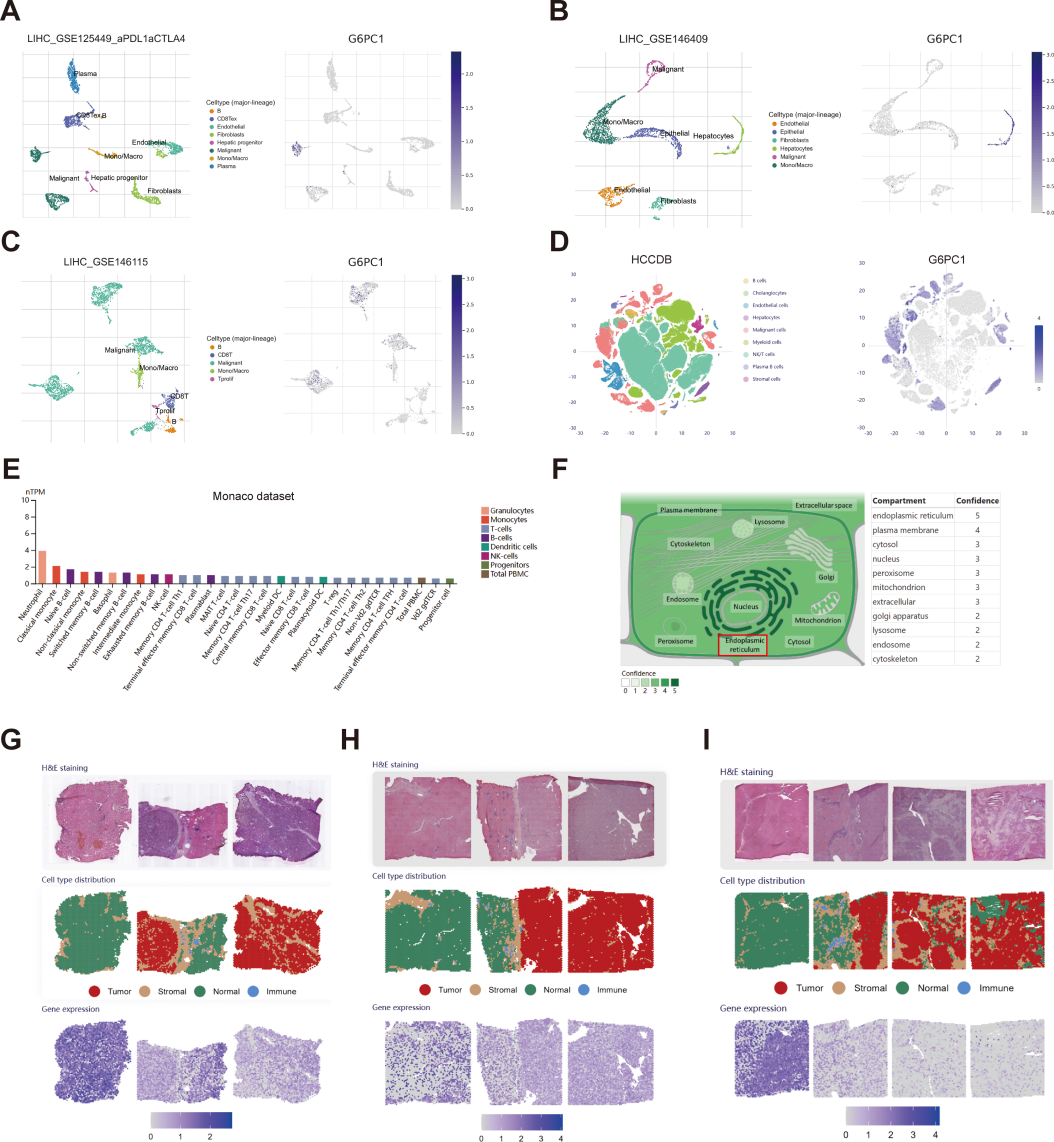
Single-cell and spatial transcriptome analysis of G6PC1. **(A–E)** The cell types and distribution of G6PC1 in different cells types in the GSE125449 **(A)**, GSE146409 **(B)**, GSE146115 **(C)**, HCCDB **(D)**, and Monaco **(E)** datasets. **(F)** Subcellular location of G6PC1. **(G-I)** H&E staining, spatial cluster distribution, and distribution of G6PC1 in different regions in spatial transcriptome data.

To gain a deeper understanding of the distribution of G6PC1 within the HCC TME, we gathered and analyzed data on G6PC1 at both the single-cell and spatial transcriptome levels from publicly available databases. For single-cell transcriptome analysis, the distribution of various cell types across four single-cell sequencing HCC datasets sourced from the TISCH2 and HCCDB databases is illustrated in **Figures 3A–D**. G6PC1 was predominantly expressed in hepatocytes and malignant cells. Notably, we found a significantly lower level of G6PC1 expression in malignant cells compared to hepatocytes in LIHC_GSE146409 and HCCDB. We concluded that the low expression of G6PC1 in HCC cells likely contributes to the poor prognosis of HCC. Additionally, G6PC1 expression is not only discovered in hepatocytes and malignant cells but also in various immune cells. The specificities of immune cell types in liver tissues were confirmed using the Monaco dataset, which revealed that neutrophils, classical monocytes, and naive B cells exhibited significantly high levels of G6PC1 expression (**Figure 3E**), indicating that G6PC1 may be involved in immune regulation, which may influence immunotherapy response. Based on data obtained from the GeneCards database, we found that G6PC1 is localized in the endoplasmic reticulum (**Figure 3F**). In terms of spatial transcriptome analysis, the hematoxylin and eosin staining and spatial cluster distribution of three HCC patients, retrieved from the HCCDB database, are depicted in **Figures 3G–I**. G6PC1 exhibited higher expression in the normal tissue compared to the tumor, stromal, and immune regions in all three HCC patients. This finding is consistent with our previous single-cell analysis results and further supports the notion that low G6PC1 expression may be associated with poor prognosis in HCC at the spatial transcriptomics level.

### 3.4 Mutation and methylation status of G6PC1

**Figure 4:**
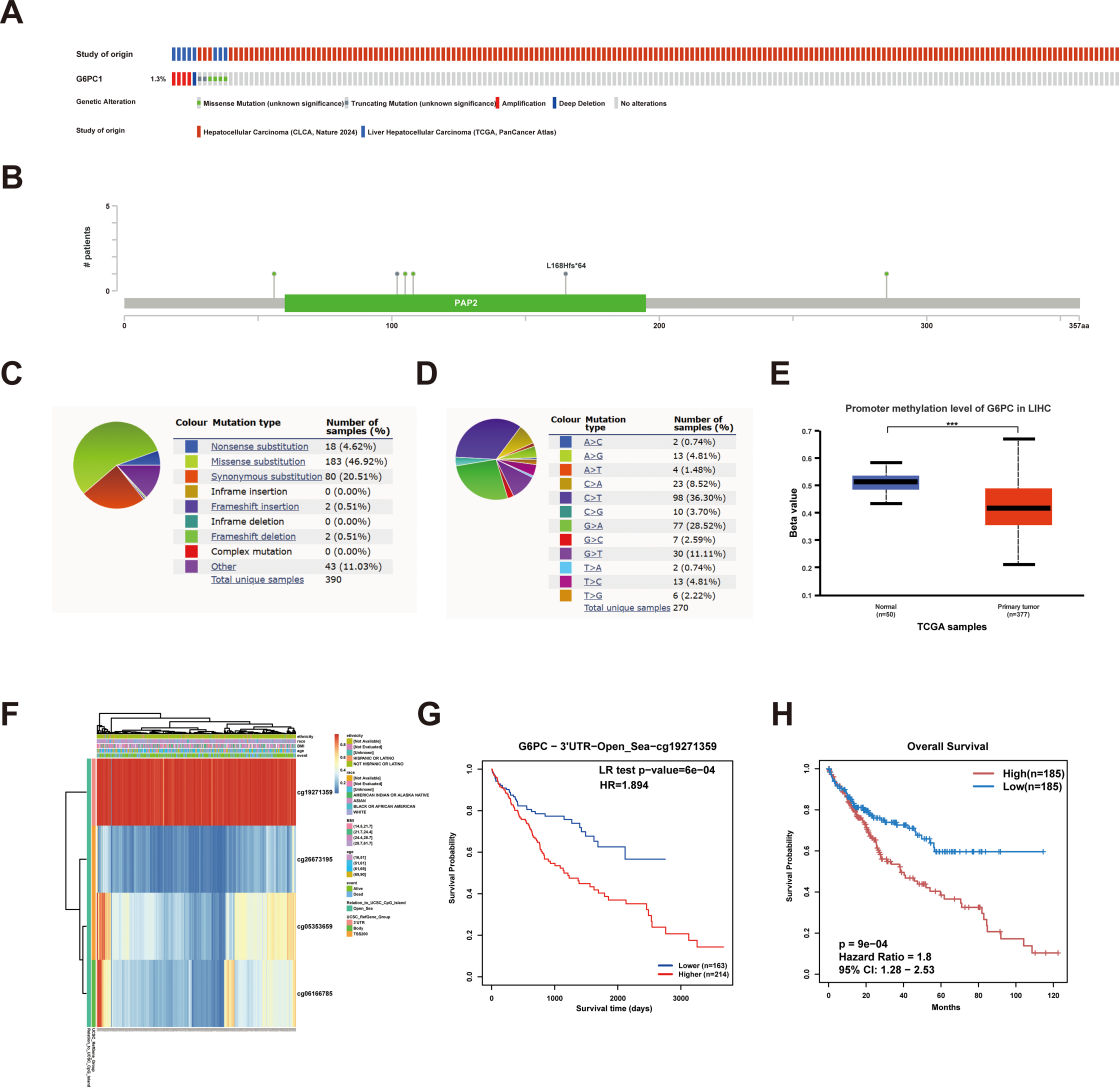
G6PC1 mutations and DNA methylation in HCC. **(A, B)** Mutation levels of the G6PC1 in cBioPortal OncoPrint. **(C)** Types of G6PC1 mutations identified using COSMIC database; **(D)** Alteration frequency of G6PC1 identified by COSMIC database. **(E)** G6PC1 methylation levels in HCC form the UALCAN database. **(F)** Correlation between G6PC1 mRNA expression level and methylation level form the MethSurv database. **(G, H)** Correlation between G6PC1 methylation level and prognosis of HCC. P values were shown as: ***P< 0.001.

Genetic mutations represent a primary etiological factor in carcinogenesis (44). In this investigation, we comprehensively characterized G6PC1 genomic alterations, including mutational profiles and CNVs, across two datasets (n=866) from cBioPortal. Our analysis revealed that G6PC1 exhibited missense mutations, amplifications, and deep deletions at an overall frequency of 1.3% (**Figures 4A, B**), corresponding to 13 mutations per 1,000 samples. COSMIC database analysis showed that missense mutations accounted for 46.92% of variants, while synonymous mutations represented 20.51% (**Figure 4C**). The most common substitution was C>T (36.30%), followed by G>A (28.52%) and G>T (11.11%) (**Figure 4D**). This indicated an absence of an association between G6PC1 mutations and HCC patient prognosis (**Supplementary Figures S1A-C**). DNA methylation analysis using UALCAN demonstrated significantly reduced G6PC1 promoter methylation in HCC tissues compared to normal liver controls (p<0.001) (**Figure 4E**). A thorough analysis of the DNA methylation status of the G6PC1 gene and the prognostic significance of CpG islands within this gene was performed using the MethSurv and SMART databases. The findings revealed that most CpG sites were hypomethylated, with the exception of cg19271359 (**Figure 4F**). Notably, hypermethylation of cg19271359 was linked to an unfavorable prognosis in HCC patients (**Figures 4G, H**).

### 3.5 Expression of G6PC1 is regulated by HNF4A, FOXA1, FOXA2, and RXRA

**Figure 5:**
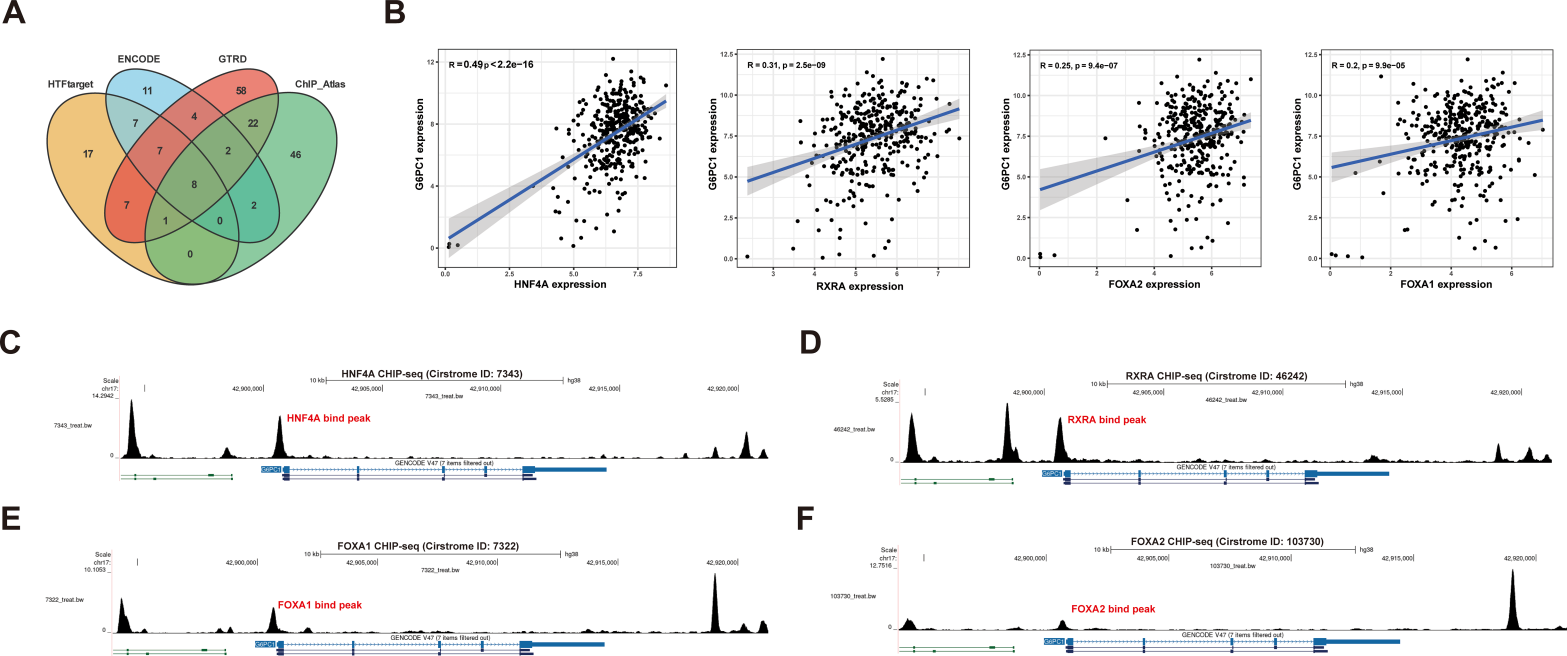
Transcription Factor Prediction. **(A)** Eight potential transcription factors were predicted through four databases. **(B)** Correlation analysis in TCGA-LIHC cohort. The CHIPseq data of HNF4A **(C)**,RXRA**(D)**, FOXA1 **(E)**, and FOXA2 **(F)** from Cistrome was visualized using the UCSC Genome Browser.

To investigate the upstream mechanisms underlying G6PC1 dysregulation, we performed TF prediction analysis. Integration of four bioinformatics databases identified eight candidate TFs (FOXA1, FOXA2, HNF4A, HNF4G, JUND, RXRA, SP1, and YY1) (**Figure 5A**). Subsequent correlation analysis revealed a significant positive association between G6PC1 and four TFs (HNF4A, FOXA1, FOXA2, and RXRA) at the transcript level in both normal and HCC tissues from the TCGA dataset (**Figure 5B**), whereas the remaining four TFs exhibited weak or nonsignificant correlations with G6PC1. Based on these findings, we focused on HNF4A, FOXA1, FOXA2, and RXRA for further analysis. Cistrome database examination of ChIP-seq data for these four TFs confirmed binding sites within the G6PC1 promoter region (**Figures 5C–F**), indicating potential direct transcriptional regulation. These results suggest that HNF4A, FOXA1, FOXA2, and RXRA may directly modulate G6PC1 expression, thereby influencing its transcriptional activity.

### 3.6 Functional analysis of G6PC1-associated genes in HCC

**Figure 6:**
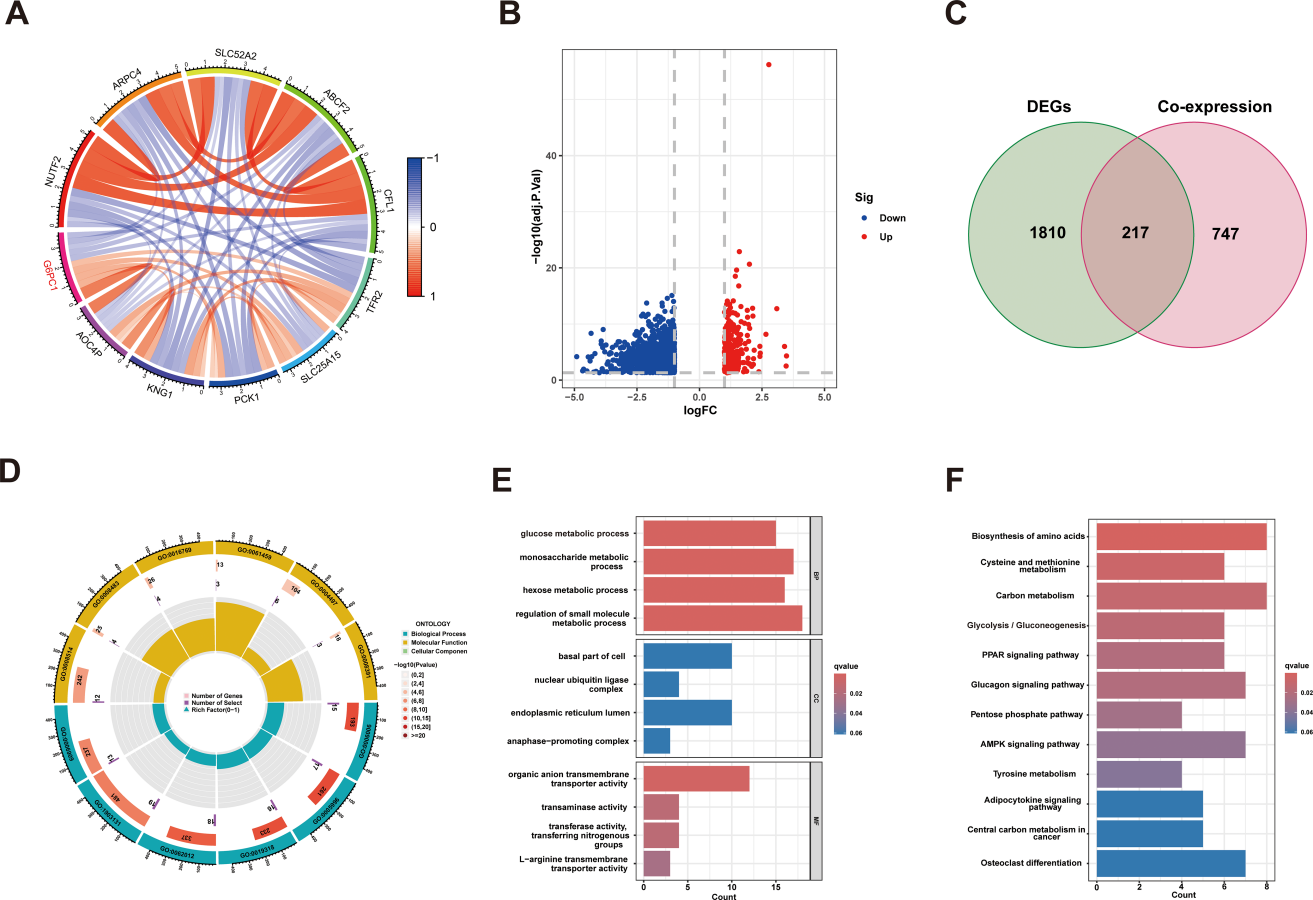
G6PC1 associations with other genes in HCC. **(A)** Circle diagram showing co-expression of genes with G6PC1. **(B)** Volcano plot of G6PC1^low^ and G6PC1^high^ differential genes. **(C)** Venn diagram showing overlap across G6PC1-coexpressed genes and DEGs. **(D, E)** GO enrichment analysis of 217 overlapping genes. **(F)** KEGG enrichment analysis of 217 overlapping genes.

To investigate the biological process, cellular component, molecular function, and metabolic pathway enrichment of G6PC1 in HCC, we analyzed co-expressed genes from TCGA data using R software. This identified 964 genes significantly correlated with G6PC1 (P<0.001, |r|≥0.4), including 330 positively and 634 negatively correlated genes (**Figure 6A**). The correlation analyses between G6PC1 and its top five positively and negatively co-expressed genes in HCC are presented in **Supplementary Figures S2A, B**. Using median G6PC1 expression as a threshold, HCC patients were stratified into high- and low-expression groups, revealing 2,027 DEGs (P<0.05, |log2FC|≥1) between these groups (**Figure 6B**). Intersection analysis yielded 217 key genes common to both co-expressed and DEG datasets (**Figure 6C**). These findings may guide future studies on the functional interactions of G6PC1 in HCC. Moreover, GO enrichment analysis demonstrated significant involvement of these genes in glucose metabolism-related processes, including glucose metabolic process, monosaccharide metabolic process, hexose metabolic process, and small molecule metabolic process (**Figures 6D, E**). KEGG pathway analysis further revealed enrichment in metabolic pathways, particularly glycolysis/gluconeogenesis (**Figure 6F**). These findings suggest G6PC1 plays a central role in metabolic reprogramming in HCC.

### 3.7 G6PC1 expression is associated with immune infiltration

**Figure 7:**
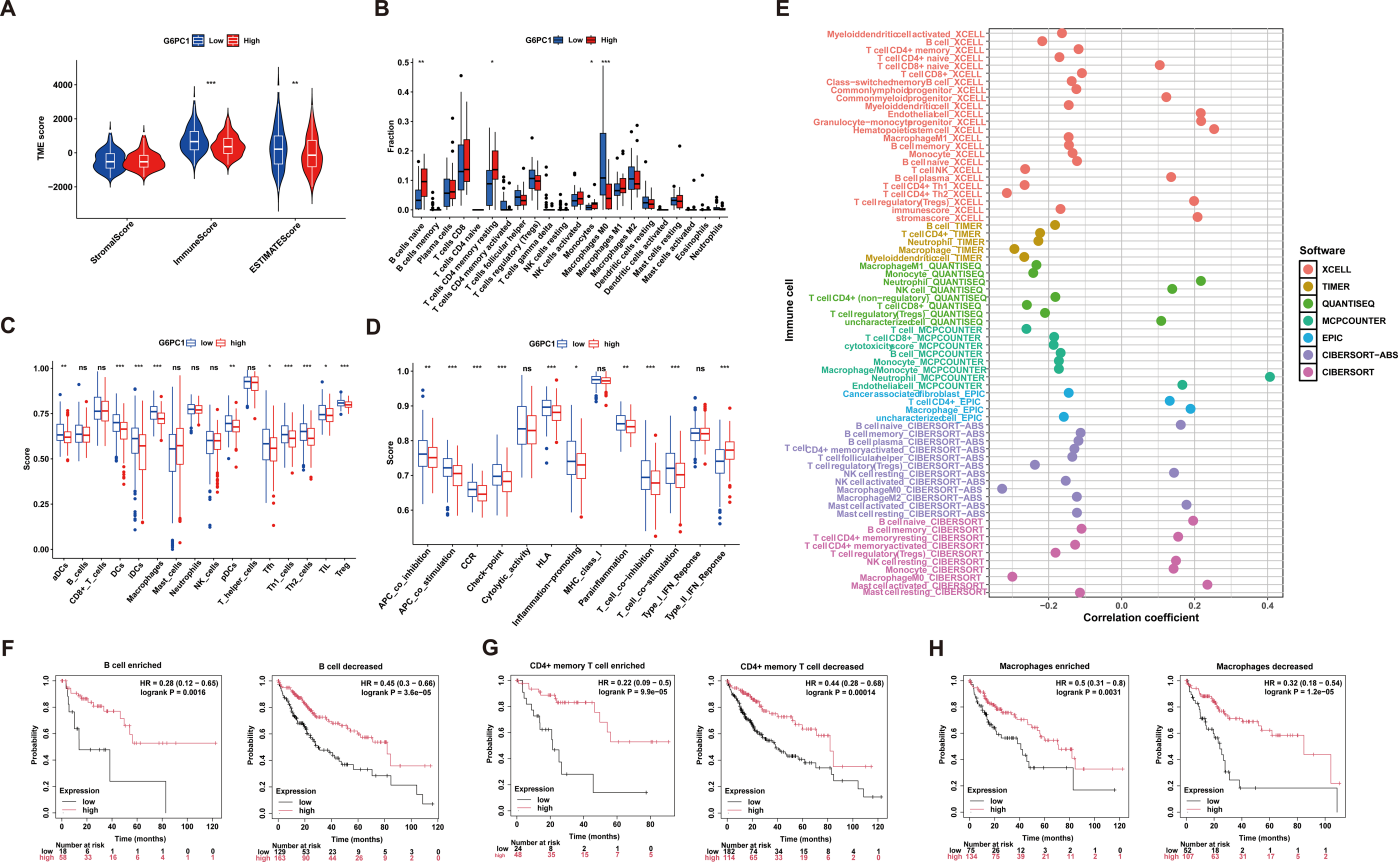
Correlation of G6PC1 expression with immune infiltration. **(A)** Differences in immune-, stromal-, and ESTMATE scores between G6PC1^low^ and G6PC1^high^ groups are shown. **(B)** Degree of immune infiltration of different immune cells in G6PC1^low^ and G6PC1^high^ groups. The scores of 16 immune cells and **(C)** 13 immune-related functions **(D)** were showed in boxplots assessed by ssGSEA algorithm. **(E)** Correlations of G6PC1 expression with immune infiltration level in HCC. Relationship between G6PC1 expression and prognosis of HCC patients based on B cell **(F)**, CD4+ memory **(G)**, and macrophages **(I)** levels. P values were shown as: ns, not significant; *P < 0.05; **P < 0.01; ***P< 0.001.

Tumorigenesis and progression are intrinsically linked to immune system interactions. Using the ESTIMATE algorithm, we evaluated immune, stromal, and ESTIMATE scores across tumors and their association with G6PC1 expression in HCC. Notably, the G6PC1-low group demonstrated significantly higher immune and ESTIMATE scores (**Figure 7A**), indicating enhanced immune infiltration. To characterize immune cell infiltration patterns, we employed ssGSEA and CIBERSORT analyses, revealing elevated enrichment scores for nine immune cell populations in the G6PC1^low^ group, including Macrophages M0, aDCs, dendritic cells (DCs), iDCs, pDCs, Tfh, Th1, Th2, TIL, and regulatory T cells (Treg) (**Figures 7B, C**). Additionally, we delved deeper into immune-related functional activities and found that the group with low G6PC1 expression had higher enrichment scores in most immune-related functions, such as APC co-stimulation, CCR, Check-point, HLA, and others (**Figure 7D**). Comprehensive immune profiling using seven algorithms (TIMER, XCELL, CIBERSORT abs, QUANTISEQ, MCPCOUNTER, EPIC, and CIBERSORT) demonstrated significant correlations between G6PC1 expression and most immune/stromal cell populations (**Figure 7E**). Given the established link between G6PC1 expression levels and HCC patient survival, as well as its correlation with immune cell presence, it is plausible to suggest that G6PC1 might influence HCC prognosis through interactions with specific immune cell populations. However, survival analysis revealed a significant difference in prognosis between HCC patients with low G6PC1 expression and those with high G6PC1 expression, irrespective of the immune infiltration levels of B cells, CD4+ memory T cells, and macrophages (**Figures 7F-H**). Thus, we hypothesize that G6PC1 may affect the survival of HCC patients by interacting with other immune cells.

### 3.8 Potential of G6PC1 to predict immunotherapy response in HCC

**Figure 8:**
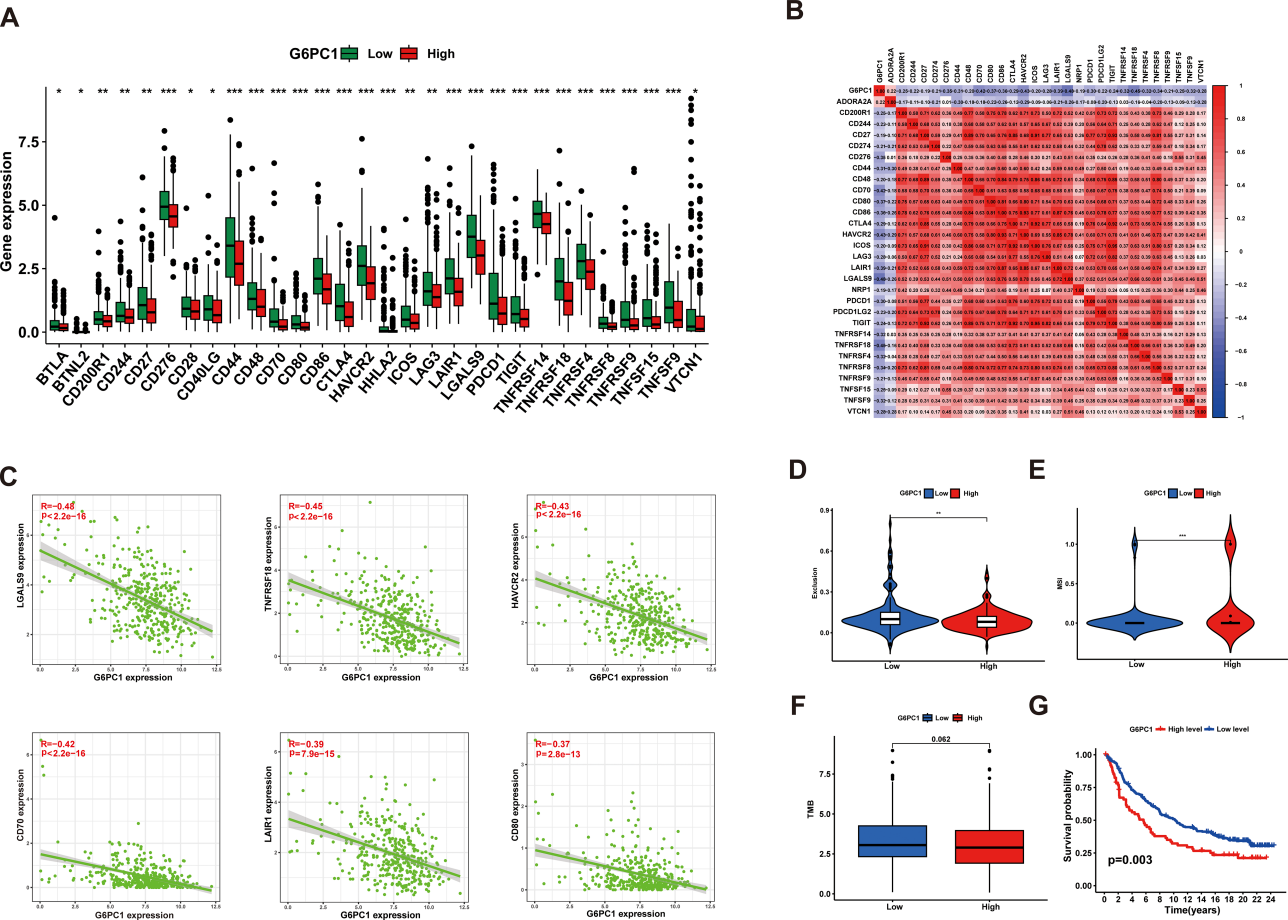
G6PC1 was helpful to predict HCC patients’ response to immunotherapy. **(A)** The difference of the immune checkpoint expression levels between the G6PC1^low^ and G6PC1^high^ groups. **(B)** Associations between levels of G6PC1 and 47 immune checkpoints. **(C)** Scatter plots of correlation between G6PC1 expression levels and six immune checkpoints. **(D)** Exclusion and **(E)** MSI scores in two G6PC1 subgroups. **(F)** The G6PC1^low^ group had the higher TMB than that of the G6PC1^high^ group. **(G)** The G6PC1 high-expression group had a poorer OS than that in the G6PC1 low-expression group. P values were shown as: *P < 0.05; **P < 0.01; ***P< 0.001.

Immune checkpoint molecules play a crucial role in mediating tumor immune escape mechanisms, and corresponding inhibitory therapies targeting these pathways have become important treatment modalities for HCC (45). Consequently, we compared the expression levels of various immune checkpoints between groups with high and low G6PC1 expression. It was found that the expression levels of several immune checkpoints, including TIGIT, LAG3, HAVCR2, and CTLA4, were higher in the G6PC1 low-expression group than in the G6PC1 high-expression group (**Figure 8A**). Additionally, we assessed the relationship between immune checkpoint expression levels and G6PC1 expression. Correlation analysis showed that G6PC1 was significantly negatively associated with most immune checkpoint expression, including LGALS9, TNFRSF18, HAVCR2, CD70, LAIR1, and CD80 (**Figures 8B, C**). This revealed that the G6PC1 low-expression group shows more of a tendency to an immunosuppressive tumor microenvironment, and might be more sensitive to immunotherapy. Given the critical role of G6PC1 in immunity, we further analyzed its impact on immunotherapy. We used MSI, TMB, and TIDE scores to predict immunotherapy outcomes. Exclusion and MSI scores were significantly higher in the low G6PC1 expression group than in the high G6PC1 expression group, indicating that the immunotherapy response rate was significantly higher in the low G6PC1 expression group (**Figures 8D, E**). Tumors with high TMB tend to respond more favorably to immunotherapy. Although there was no significant difference in TMB between patients in the high and low G6PC1 expression groups, a trend toward higher TMB scores was observed in those with low G6PC1 expression group (**Figure 8F**). To further evaluate the predictive potential of G6PC1 expression in a clinical immunotherapy setting, we conducted a study to determine if G6PC1 could forecast the response to immunotherapy and prognosis in patients with urothelial cancer receiving anti-PD-L1 treatment (IMvigor210 cohort). Interestingly, the group with low G6PC1 expression had a higher overall survival rate compared to the high-expression group (**Figure 8G**). Based on these findings, we hypothesize that patients in the low G6PC1 expression group may exhibit enhanced responsiveness to immunotherapy.

### 3.9 Drug sensitivity analysis and molecular docking validation

**Figure 9:**
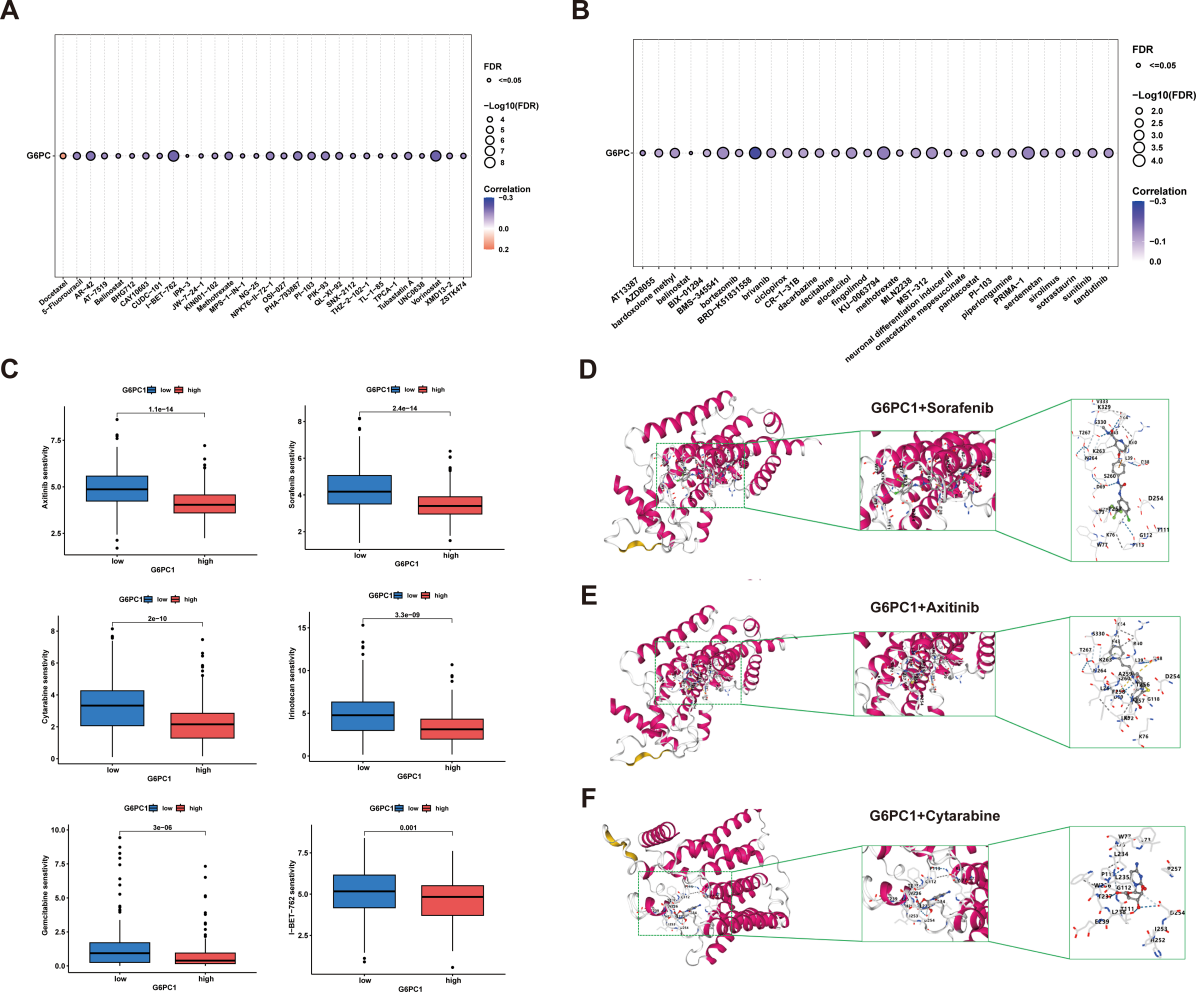
Anti-tumor sensitivity prediction and molecular docking. **(A, B)** Drug sensitivity analysis of G6PC1–1 based on the GDSC and CTRP databases. **(C)** Drug sensitivity profiles in G6PC1^low^ and G6PC1^high^ groups. **(D-F)** Axitinib, Cytarabine, and Sorafenib with G6PC1 the molecular docking structural model.

Currently, radiotherapy, chemotherapy, and immunotherapy remain crucial treatment modalities for HCC (46). To evaluate the sensitivity of anti-cancer drugs, we utilized the GSCA online platform for analysis. From the GDSC and CTRP databases, we identified the top 30 anti-cancer drugs based on their rankings and examined their associations with G6PC1 expression levels, as shown in **Figures 9A, B**. Additionally, we performed comprehensive drug sensitivity profiling in HCC, revealing that HCC patients with high G6PC1 expression exhibited greater sensitivity to axitinib, gemcitabine, irinotecan, cytarabine, sorafenib, and I-BET-762 (**Figure 9C**). Axitinib, cytarabine, and sorafenib are commonly used antitumor drugs in clinical practice, with sorafenib being the first-line treatment for advanced HCC patients. To explore the potential of G6PC1 as a drug target for HCC, we used CB-Dock2 to elucidate the interactions between the G6PC1 protein and these three antitumor drugs. As supported by previous studies, a binding energy lower than -5 kcal/mol indicates strong binding affinity (47). Our findings revealed that G6PC1 exhibited favorable binding activity with axitinib, cytarabine, and sorafenib, with binding energies of -8.1, -5.8, and -9.5 kcal/mol, respectively (**Figures 9D-F**), as detailed in **Supplementary Table S2**. These results suggest that G6PC1 may serve as a potential target for commonly used anti-cancer drugs.

### 3.10 Correlation between G6PC1 and metabolic reprogramming in HCC

**Figure 10:**
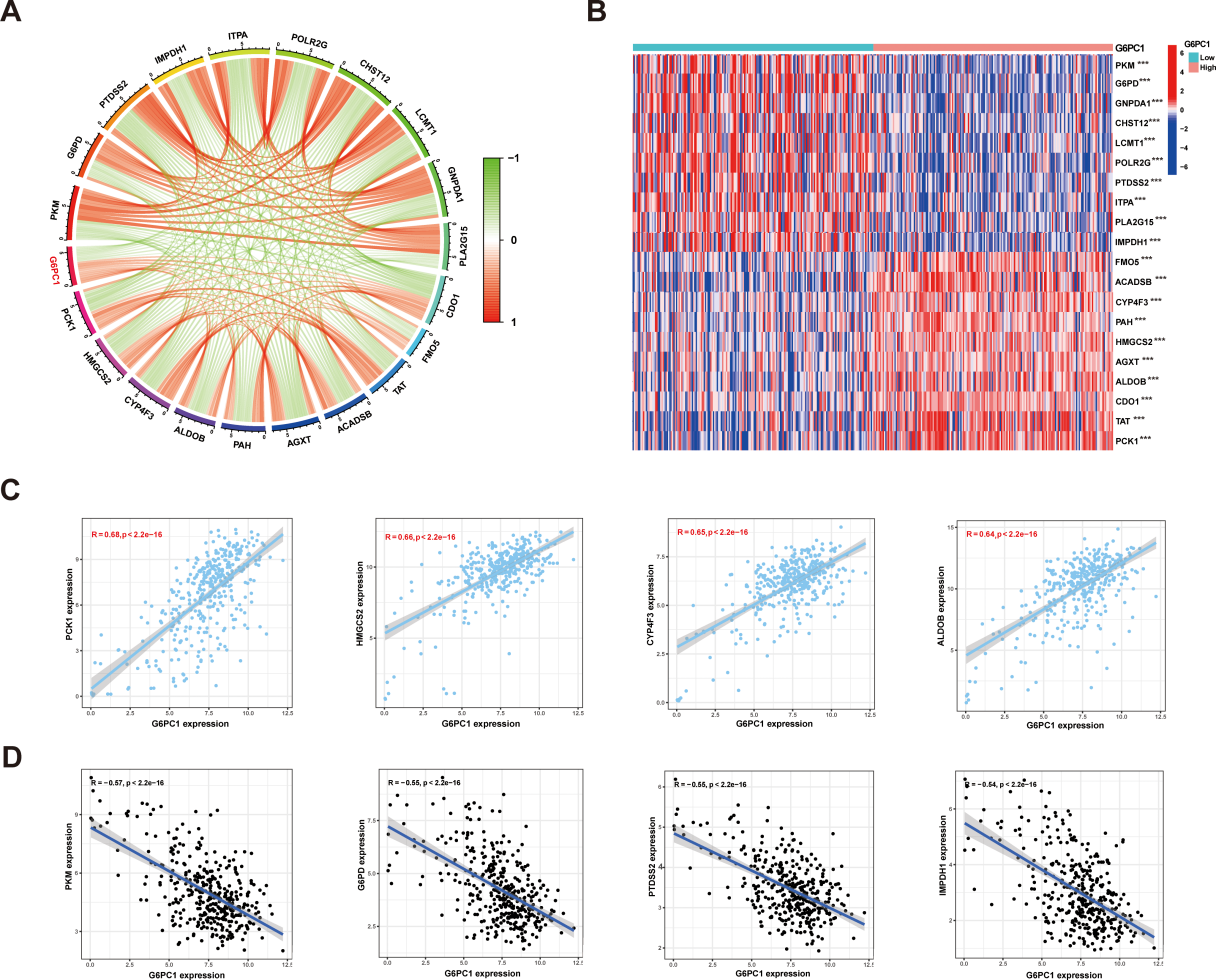
Relationships between expression of G6PC1 and metabolic related genes in HCC. **(A)** Correlations between G6PC1 and metabolic related genes using TCGA data; **(B)** Differentially expressed 20 metabolic related genes between G6PC1^low^ and G6PC1^high^ in HCC samples. **(C)** Scatterplots of G6PC1 expression against the top four positively correlated genes. **(D)** Scatterplots of G6PC1 expression against the top four negatively correlated genes. P values were shown as: ***P< 0.001.

Research has demonstrated that tumors often rely on anaerobic glycolysis to generate energy even when metabolic substrates are abundant, a phenomenon termed the Warburg Effect (48). Further investigations revealed that metabolic aberrations in tumors extend beyond the Warburg Effect, encompassing abnormalities in central metabolic pathways as well. This diversity is referred to as tumor metabolic reprogramming (49). Recent studies have highlighted that tumor metabolic reprogramming is not merely a phenotypic characteristic; it also serves as a mechanism to counteract the body’s anti-tumor immune response (50). Here, Spearman correlations were used to evaluate links between G6PC1 expression and metabolism-associated genes using the TCGA-LIHC dataset. This showed a significant association between G6PC1 expression and markers of metabolism, including PCK1, HMGCS2, CYP4F3, ALDOB, PKM, G6PD, PTDSS2, and IMPDH1 (**Figures 10A, C, D**). The TCGA-LIHC samples were divided into low and high G6PC1-expression groups and differentially expressed metabolism-related genes were identified (**Figure 10B**). It was found that these genes, including PCK1, HMGCS2, CYP4F3, and ALDOB, were expressed strongly in the high-G6PC1 group (P<0.001), while the levels of PKM, G6PD, PTDSS2, and IMPDH1 were reduced (P<0.001).

### 3.11 G6PC1 overexpression inhibits HCC cell proliferation through PKM and G6PD downregulation

**Figure 11:**
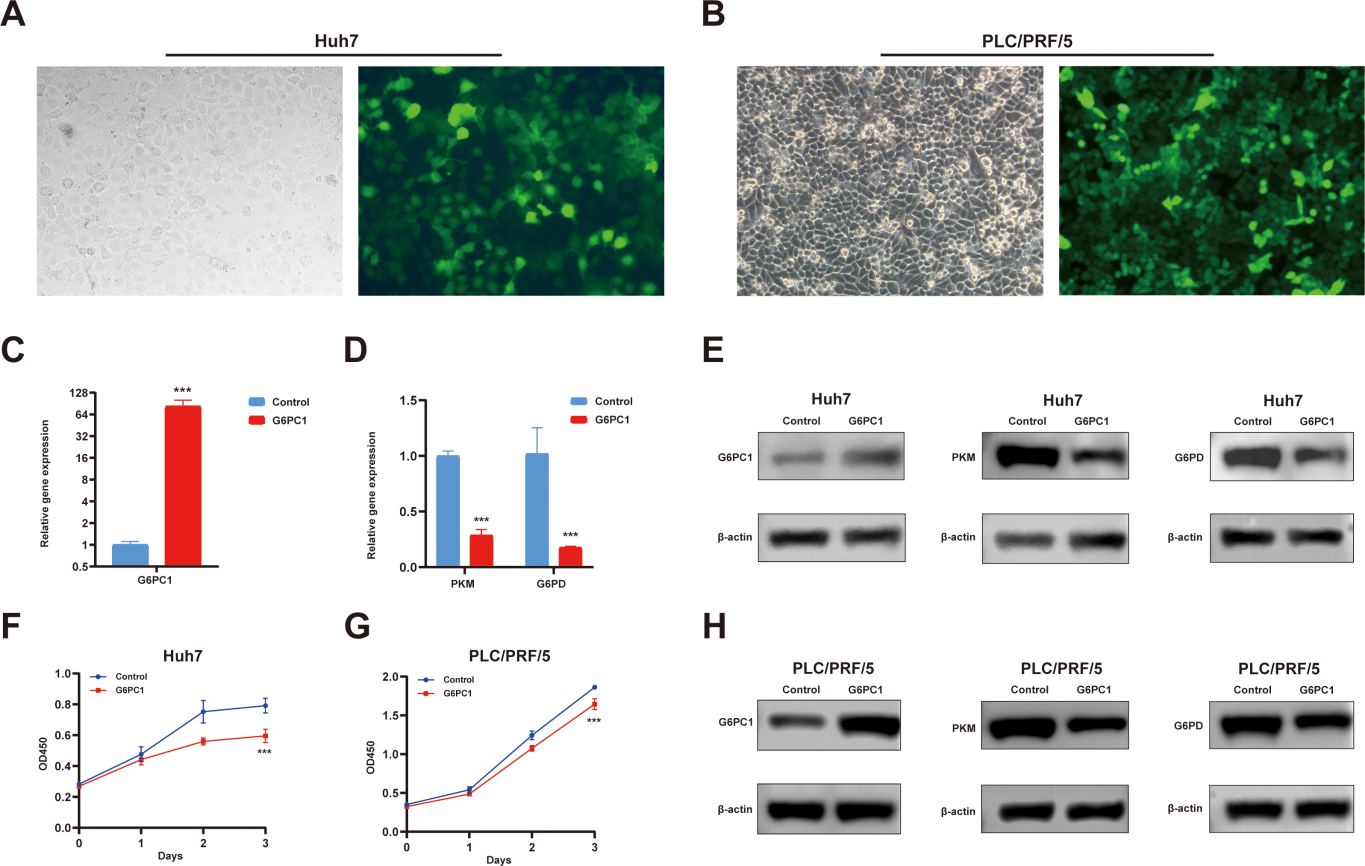
Overexpressed G6PC1 inhibits HCC cell proliferation by downregulating PKM and G6PD. **(A, B)** Green fluorescent protein signal indicating the overexpression of G6PC1. **(C, D)** mRNA expression of G6PC1, PKM and G6PD in LV-G6PC1 compared with NC group in Huh7 cells. **(E, H)** Protein expression of G6PC1, PKM, G6PD and β-actin in LV-G6PC1 compared with NC group in Huh7 and PLC/PRF/5 cells. **(F, G)** Overexpression of G6PC1 inhibits proliferation of Huh7 and PLC/PRF/5 cells. P values were shown as: ***P< 0.001.

To functionally validate previous findings, we transduced Huh7 and PLC/PRF/5 cells with lentivirus to overexpress G6PC1 (**Figures 11A, B**). Successful transduction was confirmed by GFP expression, and G6PC1 overexpression was verified by qRT-PCR (**Figure 11C**) and Western blot (**Figures 11E, H**). CCK-8 assays demonstrated that G6PC1 overexpression significantly suppressed the proliferation of Huh7 and PLC/PRF/5 cell (**Figures 11F, G**). Consistent with our bioinformatic predictions, qRT-PCR analysis revealed a significant downregulation of PKM and G6PD mRNA levels in LV-G6PC1 cells compared to LV-NC controls (**Figure 11D**). Furthermore, Western blot demonstrated that G6PC1 overexpression reduced PKM and G6PD protein expression (**Figures 11E, H**). Given that PKM and G6PD have been previously reported to promote HCC progression (51, 52), our findings suggest that G6PC1 overexpression inhibits HCC cell proliferation by downregulating PKM and G6PD.

## 4 Discussion

HCC is a highly aggressive and heterogeneous malignancy with substantial global health implications due to its elevated incidence and mortality rates (53). In this study, we innovatively integrated multi-omics analysis with experimental validation to identify G6PC1 as a potential tumor suppressor and prognostic biomarker for HCC. Additionally, we were the first to analyze and evaluate G6PC1 expression in HCC single cells using single-cell and spatial transcriptomics. Regarding the HCC TME, we extended the work of Li et al. (54). By employing seven novel algorithms to comprehensively analyze the relationship between G6PC1 and various immune cells, thereby highlighting the immunoregulatory role of G6PC1 in the TME.

The primary physiological function of G6PC1 is to catalyze the hydrolysis of G6P into glucose, thereby facilitating its release into circulation. This process is essential for maintaining blood glucose homeostasis, averting hypoglycemia, and supplying energy to peripheral organs, underscoring its pivotal role in hepatic glycogenolysis and gluconeogenesis (9). Additionally, G6PC1 contributes to renal and intestinal gluconeogenesis (55, 56).

Recent studies suggest that G6PC1 may influence not only metabolic pathways but also oncogenic processes. In several malignancies originating from non-gluconeogenic organs, such as glioblastoma, ovarian cancer, gastric cancer, and cervical carcinoma, elevated G6PC1 expression has been detected and is associated with enhanced tumor aggressiveness and metastatic potential (10, 57–59). Conversely, in cancers arising from gluconeogenic tissues, including HCC and renal cell carcinoma, G6PC1 expression is frequently suppressed, correlating with poorer clinical outcomes (12, 60). Our investigation demonstrated markedly reduced G6PC1 expression in HCC specimens compared to adjacent non-tumorous liver tissue. Furthermore, diminished G6PC1 expression was significantly associated with advanced disease stages and unfavorable survival rates. Notably, G6PC1 was expressed at low levels in malignant cells at the single-cell transcriptome level and in the tumor region at the spatial transcriptome level within the HCC TME, suggesting a potential association between G6PC1 and HCC malignancy. *In vitro* functional assays confirmed that G6PC1 overexpression inhibited HCC cell proliferation, further indicating that G6PC1 contributes to the malignant phenotype of HCC cells. Moreover, univariate Cox regression analysis and nomogram modeling further indicated G6PC1’s potential as a robust prognostic biomarker for HCC.

Cancer development is closely linked to gene mutations (61). For example, mutations in the G6PC1 have been implicated in a rare genetic condition known as glycogen storage disease type Ia. In individuals with this disorder, the accumulation of G6P is diverted into downstream metabolic pathways. This diversion results in abnormal lactate production and ectopic lipid accumulation, thereby increasing the risk of developing liver cancer (62). However, our analysis revealed that the frequency of G6PC1 gene mutation in HCC was only 1.3%, with most being missense mutations and synonymous mutations. Additionally, G6PC1 mutations were not significantly correlated with OS, PFS, and DSS in HCC. Given the limited impact of G6PC1 gene mutations on HCC prognosis, our study focused on the effects of altered G6PC1 expression levels on HCC prognosis and clinical-pathological features.

Using bioinformatics approaches, we predicted that HNF4A, FOXA1, FOXA2, and RXRA may act as transcriptional regulators of G6PC1. Among these, HNF4A, FOXA1, and FOXA2 belong to the hepatocyte nuclear factor (HNF) family, which is crucial for liver development and function (63). RXRA, a member of the NR2B nuclear receptor family, is a critical mediator of transcription regulation by diverse ligands (64). It also interacts with the liver X receptor and may influence hepatocyte differentiation. Our correlation analysis in the TCGA-LIHC cohort revealed a strong positive association between G6PC1 and these four transcription factors. Additionally, their predicted binding to the G6PC1 promoter implies potential transcriptional control over G6PC1 expression. Investigating these regulatory interactions could elucidate the molecular basis of G6PC1-mediated processes and reveal novel therapeutic strategies for HCC.

Following our investigation of upstream regulatory mechanisms, we next examined the downstream effects of G6PC1 in HCC. Intriguingly, comparative transcriptomic analysis revealed that genes differentially expressed between G6PC1^high^ and G6PC1^low^ HCC patients were predominantly enriched in metabolic pathways, particularly those governing glucose homeostasis, such as glycolysis and gluconeogenesis. These findings strongly implicate G6PC1 in the metabolic reprogramming characteristic of HCC. Metabolic reprogramming has emerged as a hallmark of cancer biology, garnering increasing attention for its role in tumorigenesis across diverse malignancies, including thyroid carcinoma, pancreatic adenocarcinoma, and gliomas (65–67). Notably, dysregulation of glucose, lipid, and amino acid metabolism has been shown to drive oncogenic progression, while metabolism-related genes have demonstrated prognostic value in multiple cancer types (68, 69). Our study identified significant associations between G6PC1 expression and two key metabolic regulators (PKM and G6PD) in HCC. Consistent with our correlation analysis, qRT-PCR and Western blot analyses confirmed that G6PC1 overexpression significantly downregulated PKM and G6PD expression in HCC cells. Both PKM and G6PD are key enzymes in the glycolysis pathway (70, 71), and contribute to HCC progression (72, 73). Our findings suggest that G6PC1 may regulate HCC development by modulating glycolysis metabolism.

Growing evidence indicates that variations in the levels of tumor-infiltrating immune cell subsets are closely associated with patient prognosis and that the pattern of immune cell infiltration can influence the efficacy of immunotherapy (74). Our immune infiltration analysis revealed that the G6PC1 low-expression group exhibited elevated levels of macrophages, DCs, and Tregs, while naive B cells, monocytes, and resting memory CD4 T cells were less prevalent in this group. These findings underscore the complexity of the tumor microenvironment in HCC. In the immune escape mechanisms of HCC, Tregs inhibit the cytotoxic activity of immune cells against HCC cells through multiple pathways, thereby facilitating immune evasion by HCC cells (75). Monocytes, which are abundant in the TME, are known to promote tumor progression through angiogenesis, metastasis, drug resistance, and immune evasion (76). DCs play a crucial role in initiating and regulating antitumor immunity, making them attractive targets for immunotherapy (77). Therefore, tumors with low G6PC1 expression harbor both immune-activating cells and immunosuppressive cells. Our study highlights the potential immunoregulatory role of G6PC1, suggesting it may serve as an indicator of immune cell activity or functional status within the TME. Given the established efficacy of immunotherapy in advanced HCC (78), we assessed the predictive value of G6PC1 in treatment response. Notably, patients with reduced G6PC1 expression exhibited significant upregulation of immune checkpoint molecules, including CTLA-4, HAVCR2, LAG3, and TIGIT. CTLA-4 is a well-established target in immune checkpoint and cancer immunotherapy. Studies have shown that CTLA-4 antibodies can effectively block the interaction between CTLA-4 and CD80/86 on T cells and antigen-presenting cells, thereby enhancing immunotherapy (79). HAVCR2 induces T cell exhaustion in cancer and chronic viral infections, and its expression in monocytes and tumor-associated macrophages is closely associated with higher HCC tumor grade and lower patient survival rates (80). Overall, our findings suggest that HCC patients with lower G6PC1 expression exhibit enhanced tumor immune suppression, implying potential responsiveness to immunotherapy.

While our study provides significant insights into G6PC1’s role in HCC, several limitations should be acknowledged. First, our findings primarily derive from retrospective bioinformatics analyses of existing datasets, which inherently limit their clinical applicability. Prospective clinical studies with larger, diverse patient cohorts will be essential to validate G6PC1’s potential as a clinical biomarker or therapeutic target. Second, while we have identified associations between G6PC1 and key metabolic pathways, the precise molecular mechanisms through which G6PC1 influences HCC pathogenesis remain to be fully elucidated. Third, this study did not investigate the molecular mechanisms mediating the crosstalk between distinct hepatocyte subsets and immune cells, which represents an important direction for future research. Future studies should explore the molecular mechanisms governing hepatocyte-immune cell interactions.

## 5 Conclusion

Our integrated investigation reveals that G6PC1 is a promising prognostic biomarker and could inhibit the cells’ proliferation in HCC, with its downregulation strongly associated with adverse clinical outcomes. Through multi-omics characterization and experimental validation, we have established the clinical significance of G6PC1 in HCC and elucidated its potential roles in HCC pathogenesis, including oncogenic mechanisms, metabolic reprogramming, and immune regulatory role. These findings position G6PC1 as both a robust prognostic biomarker and a potential predictor of immunotherapy efficacy in HCC management. They provide new insights into the role of G6PC1 in HCC and offer a potential therapeutic target. Further research is required to determine the clinical utility of G6PC1 as a biomarker and to guide treatment in HCC patients.

## Data Availability

The original contributions presented in the study are included in the article/**Supplementary Material**. Further inquiries can be directed to the corresponding author.
