# Supplementary material for: G6PC1 expression as a prognostic biomarker associated with metabolic reprogramming and tumor microenvironment in hepatocellular carcinoma
Source: Front Immunol. 2025 Aug 1;16:1623315. doi: 10.3389/fimmu.2025.1623315 (PMC12354593; doi:10.3389/fimmu.2025.1623315)
Supplement: Supplementary file 1 [file DataSheet1.docx]

**Supplementary Table S1. The sequences used in this study**

| Gene | Primers Sequences |
| --- | --- |
| G6PC1 | F: TGTGGTTGGGATTCTGGGCTGTGCA  R: TGCTGTGGATGTGGCTGA |
| G6PD | F: CTACCGCATCGACCACTACC  R: TGTTGTCCCGGTTCCAGATG |
| PKM | F: ATGTCGAAGCCCCATAGTGAA  R: TGGGTGGTGAATCAATGTCCA |
| β-ACTIN | F:CACCCAGCACAATGAAGATCAAGAT  R:CCAGTTTTTAAATCCTGAGTCAAGC |

**Supplementary Table S2. Drug prediction of G6PC1**

| **Genes** | **Drug name** | **Molecular formula** | **Molecular weight (g/mol)** |  | **binding energy (kcal/mol )** |
| --- | --- | --- | --- | --- | --- |
| G6PC1 | Axitinib | C_22_H_18_N_4_OS | 386.5 |  | -8.1 |
| G6PC1 | Cytarabine | C_9_H_13_N_3_O_5_ | 243.22 |  | -5.8 |
| G6PC1 | Sorafenib | C_21_H_16_CIF_3_N_4_O_3_ | 464.8 |  | -9.5 |
