# Supplementary material for: G6PC1 expression as a prognostic biomarker associated with metabolic reprogramming and tumor microenvironment in hepatocellular carcinoma
Source: Front Immunol. 2025 Aug 1;16:1623315. doi: 10.3389/fimmu.2025.1623315 (PMC12354593; doi:10.3389/fimmu.2025.1623315)
Supplement: Supplementary file 2 [file DataSheet2.docx]

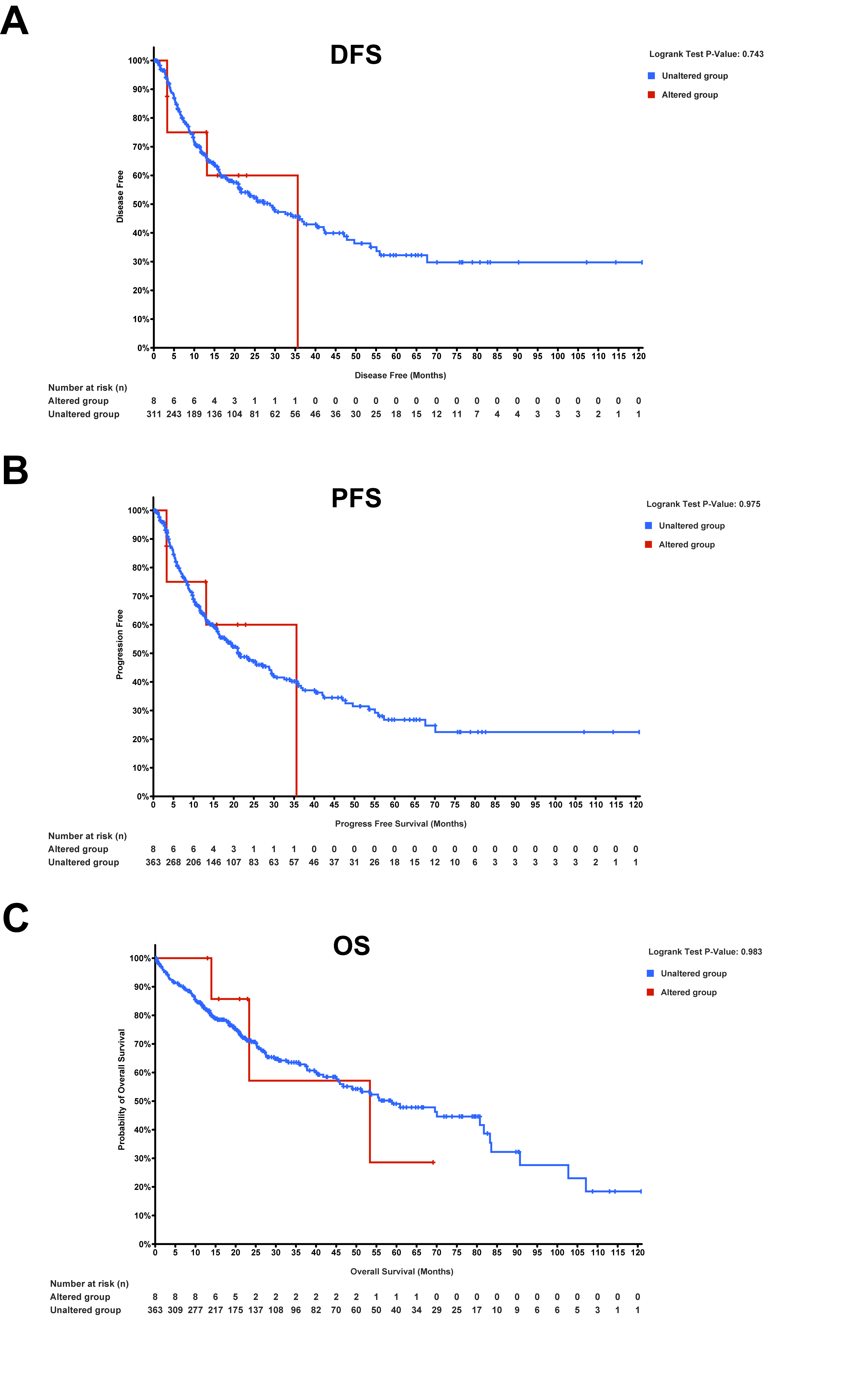


**Supplementary Figure S1.** Potential correlation between G6PC1 mutation status and survival analysis of HCC using the cBioPortal tool. Potential correlation between G6PC1 mutation status and disease-free survival (Log rank test P=0.743) (A); progression-free survival (Log rank test P=0.975) (B); and overall survival (Log rank test P=0.983) (C) of HCC using the cBioPortal tool, respectively. HCC, hepatocellular carcinomas; DFS, disease-free survival; PFS, progression-free survival; OS, overall survival.





**Supplementary Figure S2.** Correlations between G6PC1 and its top five positively and negatively co-expressed genes in HCC. (A) Five positively co-expressed genes. (B) Five negatively co-expressed genes.
